# Assessment of Domestic Pigs, Wild Boars and Feral Hybrid Pigs as Reservoirs of Hepatitis E Virus in Corsica, France

**DOI:** 10.3390/v8080236

**Published:** 2016-08-20

**Authors:** Ferran Jori, Morgane Laval, Oscar Maestrini, François Casabianca, François Charrier, Nicole Pavio

**Affiliations:** 1 UPR AGIRS (Integrated Animal Risk Management Unit), CIRAD (International Agricultural Research Center for Development), Campus International de Baillarguet, Montpellier 34398, France; 2 Department of Animal Science and Production, BUAN (Botswana University of Agriculture and Natural Ressources), Private Bag 0037, Gaborone, Botswana; 3 Laboratoire de Recherche sur le Développement de l’Elevage (LRDE), Institut National de la Recherche Agronomique (INRA), Corte 20250, France; mlaval@etu.isara.fr (M.L.); oscar.maestrini@inra.fr (O.M.); francoiscasabianca@inra.fr (F.C.); francois.charrier@inra.fr (F.C.); 4 Animal Health Laboratory, UMR 1161 Virology, ANSES, Maisons-Alfort 94706, France; nicole.pavio@anses.fr; 5 UMR 1161 Virology, INRA, Maisons-Alfort 94706, France; 6 UMR 1161 Virology, National Veterinary School, PRES University Paris 12, Maisons-Alfort 94706, France

**Keywords:** hepatitis E virus, wild boar, domestic pig, hybrids, Corsica, *Sus scrofa*, zoonoses

## Abstract

In Corsica, extensive pig breeding systems allow frequent interactions between wild boars and domestic pigs, which are suspected to act as reservoirs of several zoonotic diseases including hepatitis E virus (HEV). In this context, 370 sera and 166 liver samples were collected from phenotypically characterized as pure or hybrid wild boars, between 2009 and 2012. In addition, serum and liver from 208 domestic pigs belonging to 30 farms were collected at the abattoir during the end of 2013. Anti-HEV antibodies were detected in 26% (21%–31.6%) of the pure wild boar, 43.5% (31%–56.7%) of hybrid wild boar and 88% (82.6%–91.9%) of the domestic pig sera. In addition, HEV RNA was detected in five wild boars, three hybrid wild boars and two domestic pig livers tested. Our findings provide evidence that both domestic pig and wild boar (pure and hybrid) act as reservoirs of HEV in Corsica, representing an important zoonotic risk for Corsican hunters and farmers but also for the large population of consumers of raw pig liver specialties produced in Corsica. In addition, hybrid wild boars seem to play an important ecological role in the dissemination of HEV between domestic pig and wild boar populations, unnoticed to date, that deserves further investigation.

## 1. Introduction

In several industrialized countries, the zoonotic origin of hepatitis E virus (HEV) infection has been demonstrated [1,2,3,4]. Pig, wild boar and deer are considered as the main free-ranging animal reservoirs of human contaminations [1] but HEV is also present in many other mammal species [5,6]. The first confirmed cases of zoonotic transmission were described in Japan in the early 2000s, after consumption of infected raw deer meat (*Cervus nippon*) and undercooked wild boar meat [7]. Subsequently, several documented cases of HEV infection after consumption of pig, wild boar or deer meat have been reported in Japan and in Europe [1,4,8].

In pig farms from continental France, a previous countrywide study had estimated a seroprevalence of 65% at farm level with higher values in the North-West of the country [9] where the majority of the pig farms are located. On the other hand, a North-to-South increasing gradient has been observed in wild boar seroprevalence (7.2% to 22.7%) [10]. The virological prevalence of HEV in pig livers in continental France is estimated at 4% [9]. Recently, grouped cases of hepatitis E have been described in France after consumption of a Corsican cured meat specialty known as “*ficatelli*” produced with pig livers from Corsica or continental France, and traditionally consumed raw or grilled [11,12]. Furthermore, *ficatelli* sold and consumed in Corsica have been found positive for HEV [13].

Data on the prevalence of HEV in human patients in Corsica is to date limited to a preliminary survey in small sample of 82 individuals, which suggests that the human prevalence of HEV is very high (an average of 73%) and affects diverse population strata such as pig farmers or forestry workers [14]. This was confirmed by a recent survey among adult French blood donors which showed that the overall prevalence of anti-HEV immunoglobulin (Ig)G antibodies in Corsica was almost 40%, the second highest in the country [15].

While the presence of HEV RNA and antibodies in pig and wild boar populations has been studied in continental France, no data is available on the status of potential common reservoirs such as domestic pig and wild boar populations from Corsica with regards to HEV infection. In Corsica, domestic pigs are commonly raised in semi-open or open spaces, the latter being particularly widespread and anchored in cultural traditions from the island [16,17]. This kind of breeding system allows frequent interactions with wild boars, equally widespread in the Corsican ecosystem, resulting in a free circulation of feral wild boar × domestic pig hybrids [18,19] that are perfectly distinguishable from pure bred Eurasian wild boars [20]. The aim of this study was to determine viral and serological prevalence of HEV in domestic pigs, wild boars and its hybrids in Corsica. Results are discussed in the light of the widespread traditional habits of pig farming, hunting and producing, selling and consuming raw liver cured meat and the potential zoonotic transmission resulting from these practices in Corsica and continental France.

## 2. Materials and Methods

### 2.1. Study Area

The study was carried out in selected regions from the Department of Haute Corse and from the Department of Corse du Sud. A patchy distribution of pig farms and hunting areas is widespread in these regions.

The center of the island is occupied by highland habitats which form a single chain of 21 summits more than 2000 meters (6600 ft) above sea level. The slope of the terrain varies significantly from area to area. The vegetation in the pastures reflects the influence of both Mediterranean and mountain climates, with scrub (*maquis*), a mixture of rapidly growing evergreen herbs, bushes and small trees, holm oak (*Quercus ilex*), cork oak (*Quercus suber*), and olive trees up to 600 m, chestnut trees between 600 and 1000 m, and mostly grass above 1000 m above sea level. Oak and chestnut pastures are usually utilized to feed pigs in autumn [17], whereas grass mountain pastures are only used in summer. Pigs reared under the traditional breeding systems are mostly from the local breed called “Nustrale” [17].

### 2.2. Sampling Strategy

**Figure 1:**
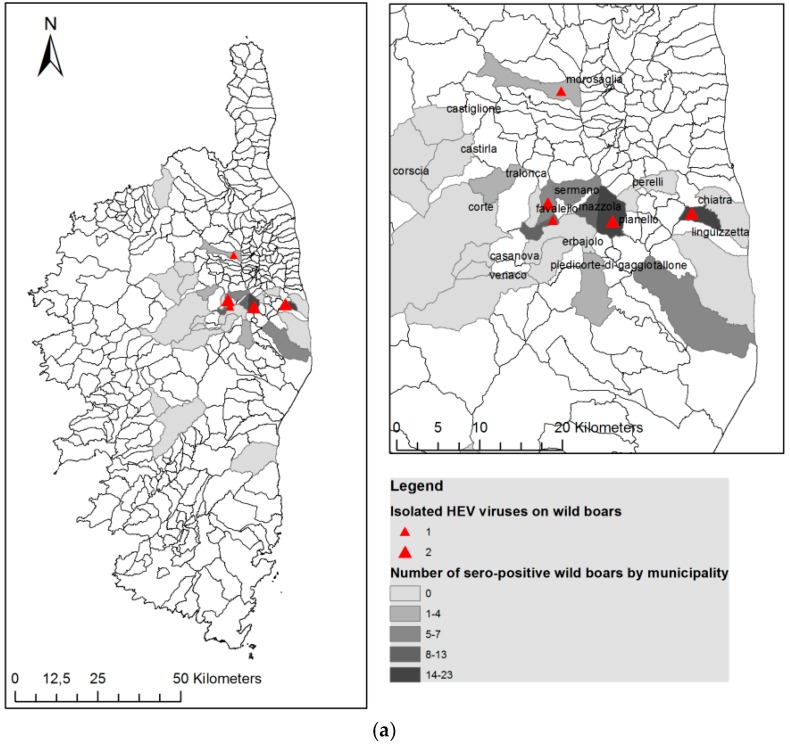

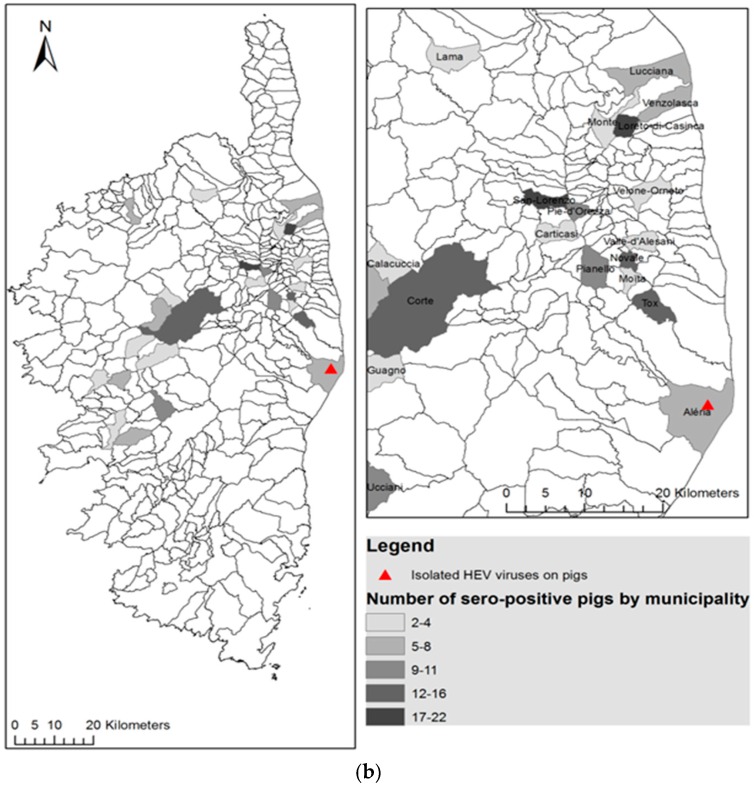
Map of Corsica showing the overall distribution of seropositivity to hepatitis E virus (HEV) antibodies and the locations where HEV was isolated (**a**) in wild boar and (**b**) in domestic pigs.

The sampling strategy was purposive and mainly driven by practical opportunities obtaining wild boar and domestic pig samples. In this manner, wild boar samples were obtained from 28 locations distributed in Northern (*n* = 25) and Southern Corsica (*n* = 3), where groups of hunters collected samples from hunted boars (Figure 1). All the samples were obtained from certified hunters with hunting license after receiving training for sample collection by a qualified technician from INRA- LRDE.

Domestic pigs were sampled from two official abattoirs in Northern and Southern Corsica (Figure 1). Ten visits to the abattoir were organized between December 2013 and February 2014, which is the usual period of pig slaughtering in the traditional pig farming system [17]. The name, origin and breeding system of the pig farm (close, open or semi-open) was recorded.

#### 2.2.1. Wild Boars Samples

A total of 370 wild boars were shot during three hunting seasons between 2009 and 2013. The sex, age and breed were recorded. The age was determined according the tooth eruption pattern [21], and subdivided into three categories of age (young, <14 months; sub-adult, 15 months–25 months; adult, >26 months) according to previous publications [22]. For the assessment of wild boar phenotypes, the presence of different colors other than black or dark brown in the coat and the shape and length of the ears were considered as indicators of hybridization with domestic pigs [20].

Among the 370 blood samples collected just after death, only 346 were suitable for analyses. These samples included 50% of young animals, 21% of sub-adults and 29% of adults. Eighty-two percent of the animals were classified as having a pure wild boar phenotype while 18% were classified as hybrid animals. The sex ratio was 1:1. During dressing of the carcasses, a small piece of hepatic tissue (*N* = 166) was taken on the inner face of the liver, near the hepatic vessels or the bile (*N* = 186) was extracted from the gallbladder and stored at −80 °C. After centrifugation at 4500 rpm for 5 min (Wifug centrifuge, Labor 50, 10219, Bradford, UK), the serums, from all campaigns, were kept at 4 °C for a maximum of 15 days, or frozen at −20 °C until processing.

#### 2.2.2. Pigs Samples

Serum, bile and/or liver were collected from 208 domestic pigs originating from 30 farms. Due to the low number of animals per farm (median = 160, interquartile range (IQR): 100–212), the batch size of pigs sampled per day at the abattoir varied from one to 18 pigs (median = 5, IQR: 4–8.5). For all animals, relevant information on age, sex, farmer and type of farming was collected. The farms raising pigs indoors (with or without outdoor access) were classified as a closed breeding system (13% of the sample), those raised outdoor but on a fenced territory were classified in semi-open breeding system (17.3% of the sample) and those raised outdoors without defined boundaries were classified as open breeding system (69.8% of the sample). In two farms, the breeding system could not be classified and were excluded from the analysis. The age, determined by the official ear tag, allowed to divide the pig sample into four categories: young (<12 months) representing 5.8% of the sample, sub-adult (12–23 months) encompassing 7.3% of the sample, adult (24–35 months) present in 62.1% of the sample and old (>35 months), representing 24.7% of the pigs. The blood was obtained at the bleeding post during slaughtering and centrifuged at 4500 rpm for 5 min to keep serum. A small fragment (1 cm × 0.5 cm × 0.5 cm) of liver was cut on visceral face above the gallbladder. The gallbladder was incised and the bile obtained was stored at −20 °C with liver and serum samples until analyses. Because of a limited budget, only 24 samples (22 livers and two biles) were analyzed for RNA detection, chosen in each batch where seropositive pigs were found. Any farm with at least one seropositive animal was considered positive for HEV.

### 2.3. Serological Analysis

The detection of anti-HEV antibodies for both pigs and wild boars from the three hunting seasons was performed using the HEV ELISA 4.0v kit (MP Diagnostics, Illkirch, France) according to the manufacturer’s instructions, except the serum quantity used, 10 μL instead of 20 μL. This sandwich ELISA allows the detection of all antibody classes (IgG, IgM and IgA) and uses a recombinant antigen that is present in all HEV strains. Samples were positive when the optical density at 450 nm wavelength obtained for the sample was higher than the threshold defined as the mean for negative controls +0.3.

### 2.4. Virological Detection

RNA extraction from liver was performed manually as described in [9]. RNA extraction from bile was performed manually using the QIAamp Viral RNA extraction Mini kit (QIAGEN, Illkirch, France) according to the manufacturer’s instructions except that 200 μL of bile was used. HEV RNA detection in bile or liver samples was performed using real-time quantitative RT-PCR as described in previous literature [23].

### 2.5. Statistical Analyses

All the analyses were implemented with Epi Info (CDC, Atlanta, GA, USA). A chi-squared (Fisher’s exact) test was used to compare the potential association between seropositive wild boar and sex, age, hunting season and phenotypical appearance. The proportion of seropositive domestic pigs was equally compared according to sex and the type of breeding system. *P*-values lower than 0.05 were considered significant. Percentage of positive for RNA and HEV-antibodies detection and 95% confidence interval (CI) associated was calculated using a one-sample proportions test or an exact binomial test. Due to the small proportion of RNA positive pigs and wild boars, comparative statistical analysis was not applicable in this group of animals. The location of all the animals was recorded on city scale and the maps were created using the ArcGis Plattform 10.2 (ESRI, Redlands, CA, USA).

## 3. Results

### 3.1. Prevalence of HEV in Wild Boar

Anti-HEV antibodies were detected in 101 of 346 wild boar serums tested (29.2%, 95% CI = 24.5%–34.4%) and eight wild boars on 352 (2.3%, 95% CI = 1.0%–4.6%) were positive for HEV RNA after real-time quantitative (RT)-PCR. The detailed serological results in the different subgroups are reported in Table 1 and Figure 1.

**Figure 2:**
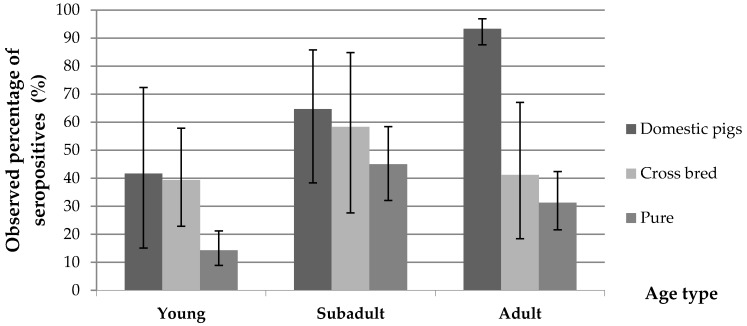
Comparison of seropositivity percentage values in domestic pigs, hybrids and pure wild boars showing a significantly higher prevalence among young hybrid animals (*p* = 0.006) when compared with pure wild boar.

A significant difference of seropositivity (*p* < 0.01) was found between wild boars characterized as hybrid species (43.6%, 95% CI = 31.0%–56.7%) when compared to animals characterized as phenotypically pure wild boar (26.1%, 95% CI = 21.1%–31.6%). This difference was consistent across hunting seasons (Table 2) and highly significant in young animals (Figure 2).

**Figure 3:**
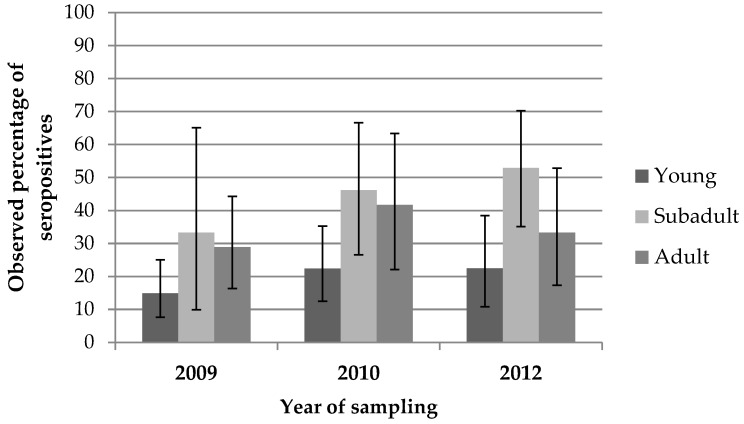
Seroprevalence values (%) in hunted wild boars (all categories) stratified by age and hunting season. Seroprevalence is lower in young animals, increases in sub-adults and drops in adults. These differences per age were consistent across the seasons and significant in 2010 (*p* = 0.05) and in 2012 (*p* = 0.02).

Seroprevalence was also significantly different when stratifying for the hunting season (*p* < 0.04), increasing progressively from 2009 (21.4%) to 2010 (33%), and subsequently to 2012 (35.6%). Seroprevalence in the whole wild boar population sampled was significantly different when stratifying by age (*p* < 0.00001), being higher in sub-adult individuals (47.2%), followed by adults (33%), and young animals (19%). This difference was also consistent across seasons, but only significant for 2010 and 2012 (Figure 3).

HEV RNA was detected in eight young wild boars of which 75% (6/8) were also seropositive (the remaining ones were not tested because sera could not be obtained), and 40% (3/8) were classified as hybrid while the remaining one was classified as pure wild boar. They were almost evenly distributed across seasons (two in the 2009–2010 season and three in the two remaining seasons). The highest levels of prevalence were located at the East of the island, where the majority of viraemic animals were also isolated (Figure 1a).

### 3.2. Prevalence of HEV in Domestic Pigs

The overall seroprevalence for domestic pigs was 87.98% (95% CI = 82.6%–91.9%) and HEV RNA was detected in two of the 24 pig samples tested (8.3% 95% CI = 10.3%–27.0%). All the pig farms tested had at least one seropositive animal. The proportion of seropositive pigs is presented in Table 1 and Figure 1b. Differences of seroprevalence stratified by sex were not significant. However, we found apparent differences between age groups, seropositivity increasing with age, from 41.7% in young animals to 64.7% in sub-adult individuals and up to 93% and 94% in adult and old animals, respectively (Table 1). Differences between animals younger and older than 2 years were significant (*p* = 0.002). 

The prevalence was significantly smaller in animals raised in closed breeding systems than in those raised in any kind of open system (50% versus 93%, *p* = 4 × 10^−7^). The two positive pigs in which RNA virus was detected among 24 pig samples, were less than 6 months old and belonged to the same farm keeping pigs in a closed system.

## 4. Discussion

In continental France, more than 1800 cases of human infections of HEV are reported annually [24] and several studies have recognized the importance of wild and domestic pig reservoirs as a source of HEV virus [11]. A recent epidemiological study across France, showed that HEV was hyperendemic among Corsican blood donors [15]. In contrast, in Corsica, where wild boars and free ranging domestic pigs are abundant and often in close contact [18,19], no data on HEV prevalence had been produced to date. Furthermore, Corsica produces a traditional and popular food specialty with raw pork liver (*ficatelli*), which is widely consumed locally and in continental France. A large proportion of human cases detected in France are linked with consumption of this product [11,13]. In addition, hunting, which is another recognized risk factor for zoonotic HEV transmission [25], is very common in Corsica with approximately 30,000 wild boars killed annually by 200–250 hunting teams and total of 17,000 licensed hunters [26].

### 4.1. Prevalence in Domestic Pigs Reared in Open Production Systems

Most of the literature on HEV in pigs originates from intensive systems where piglets are known to become infected between eight and 12 weeks of life, after losing maternal immunity [27]. The HEV seroprevalence observed in our sample of Corsican pigs can be considered high (87.98%) and is in any case much higher than in continental France, where 31% of slaughtered pigs were seropositive for HEV during a large scale survey [9]. In our study, HEV was found present in all of the farms tested (*n* = 31), while HEV circulated in 65% of continental French farms (*n* = 186).

HEV RNA was detected in 8.3% of the liver or bile tested (*n* = 24). This value is higher than in continental settings but positive animals were sampled at less than 6 months of age and it has been demonstrated that the detection of HEV in pig livers increases in individuals slaughtered at an early age [3]. The HEV RNA prevalence estimated in the present study remains in the range of other Western countries with up to 11% of positive livers sold in grocery stores in the USA [28,29]. The two positive pig samples were collected in the same herd during 2013. Therefore, in comparison to other studies from other European countries, our work provides new information on the prevalence of antibodies in older animals reared in outdoor or open systems. In Corsica the majority of pigs are reared in open or semi-open systems and slaughtered at ages older than one year. Surprisingly, the seroprevalence detected in closed systems was significantly lower than the one detected in open systems. Despite the specificities of the different pig breeding systems, the overall seroprevalence observed in our limited sample of Corsican pigs obtained during the first year of life (41.70%), remains comparable to the one reported in other European countries [6,30,31]. A possible explanation is that despite in open systems the density of pigs is likely to be lower than in intensive pig farms, animals in open or semi-open systems tend to concentrate around food and water points reaching densities that are sufficiently high to allow regular exposure to most of the herd through the contamination of food and water. In that sense, it is likely that susceptible pigs are regularly exposed, becoming infected and producing antibodies until most of the population is exposed and immune, as shown by the high level of immunity observed after two years of age (Table 1 and Figure 2). This information suggests that if *ficatelli* are manufactured on-farm using only livers from Corsican pigs produced in extensive conditions, the risk of those livers being infected with HEV remains low. However, in practice, since the demand of this product is high all year round (and not only in winter during the slaughter period of the local breeders), Corsican livers do not suffice to produce enough the volume of *ficatelli* required by the market. This deficit is covered by large numbers of livers from pigs produced under intensive systems in continental Europe being imported into Corsica to manufacture *ficatelli*, with a higher risk of being infected with HEV. Indeed, during a previous study on HEV contamination of *ficatelli* from Corsica [13], high sequence identities, as high as 97 to 99%, were observed between HEV from *ficatelli* (Genbank accession numbers KJ558437, KJ558440, KJ558441, KJ558442, KJ558445, KJ558448, KJ558478, KJ558481, KJ558494) and swine liver from continental France (Genbank accession numbers JF718820, JF718798, JF718817 JF718828, JF718797).

### 4.2. Prevalence in Wild Boars

The prevalence rates observed in wild boars were substantially higher than in continental France, which ranged between 7.2% to 22.7% [10] but lower than those observed in Southern Europe where densities are known to be high [31,32]. Differences in prevalence across years are unlikely to be due to different storage or laboratory conditions since all the samples were stored, managed and analyzed in a standardized way.

Several authors have described an association between densities of wild boars and HEV seroprevalence [4,31,33,34]. In our study, data on densities of wild boar were not available. However, prevalence of antibodies which was low in young animals (19%), reached its highest level in sub-adult animals (50%) and dropped again in adult animals (33%). These data show that prevalences in wild boars (all phenotypes) are lower than in domestic pig farms reared under extensive conditions, possibly due to lower densities of animals and transmission rates between individuals. The fact that HEV RNA was isolated only from young individuals suggests that infections occur during the first months of life. However, seroprevalence profiles by age (Figure 2 and Figure 3) seem to confirm that exposure to the virus does not reach the majority of the population, and suggests some loss of immunity at older stages of life. Therefore, an insufficient immunological protection could allow subsequent reinfections during the life of the wild boars as suggested by some authors [34,35,36].

### 4.3. Prevalence in Hunted Hybrid Pigs

Our study detected significant differences in the HEV seroprevalence of hybrid pigs (43.5%), compared to the values detected in phenotypically pure wild boar (26%). These differences were consistent in every hunting season (Table 2) and particularly significant during the first year of life (Figure 2). In some locations where wild boar and domestic pig activity patterns have been compared, wild boars show nocturnal habits while domestic pigs have diurnal habits and consequently direct interactions are limited [37,38]. Local hunters from Corsica confirm the same activity patterns for wild boars and domestic pigs and maintain that hybrid animals show social patterns which are more similar to domestic pigs (more diurnal) and more able to interact with the later than with pure wild boars. In addition, it is known that hybrids breed more regularly and have larger litters than pure wild boars [20,39], which explains potential higher densities in groups of hybrids and therefore, a more favorable environment for the transmission of HEV. From that perspective, and despite our genetic classification of wild boars relied purely on phenotypical characters (coat color, ear shape) and not in sophisticated genetic analyses [40], some misclassification could have occurred (particularly from some hybrid individuals being classified as “pure“). Nevertheless, our results provide a consistent indication that hybrids could play a specific epidemiological role in the dissemination of HEV and perhaps other pathogens between domestic pigs and wild boar populations. In fact, similar observations have been recently observed in the Netherlands when comparing the prevalence of *Mycoplasma hyopneumoniae* in hybrids which was higher than in pure wild boars [40]. Similarly, in China, hybrids reared in captivity showed higher prevalences of porcine reproductive respiratory syndrome (PRRS) than domestic pigs reared in similar conditions [41]. Considering that wild boar hybrids are quite widespread in many countries worldwide, their potentially distinct epidemiological role in the dissemination of pathogens between domestic pigs and wild boars deserves further investigation.

## 5. Conclusions

In conclusion, this study shows that wild and domestic pig reservoirs of HEV in Corsica are highly exposed to HEV. The phylogenetic analysis of the sequences amplified in both reservoirs was performed in parallel to this study and has shown that the two sequences of pure wild boar and domestic pig (Genbank accession number KT334196 and KT334197) shared 97.5% identity with a wild boar sequence (KT334191), suggesting that transmission between pure and hybrid forms of wild boar and domestic pigs occur in Corsica [42]. Hybrid wild boars seem to play an intermediate role in this dissemination of HEV and perhaps other pathogens between populations of domestic pigs and wild boars that requires further investigation. The high levels of prevalence detected in all varieties of *Sus scrofa* are particularly relevant in terms of public health risk, considering the frequent contacts of local stakeholders with domestic pigs, wild boars and their derived products, the local tradition of processing and consuming pork products with raw pig liver, and the popularity of hunting and extensive pig farming activities in Corsica. Moreover, there is also a non-negligible risk for the large numbers of potential consumers of pig cured or raw products visiting Corsica or consuming those products outside the island. To support this hypothesis, the phylogenetic analysis has underlined also that HEV sequences amplified in Corsican swine and wild boar have high nucleotides identities with those from *ficatelli* or human cases of hepatitis E from continental France [42]. All these activities are significant risk factors for HEV infection in human patients [15] and awareness campaigns should be organized among all potential stakeholders (farmers, hunters, butchers and *ficatelli* producers in Corsica and all *ficatelli* consumers outside the island) about the HEV infection risk incurred in each case and the need to consider potential protecting measures [25].

## Figures and Tables

**Table 1:** HEV apparent seroprevalence percentages in domestic pigs and wild boar by gender, breed, hunting season and age.

		Total Sample	Number of Positive	Seroprevalence (%)	Confidence Interval (95%)
**Wild boars**					
**Sex**	Male	173	51	29.4	22.9–37.0
	Female	173	50	28.9	22.4–36.4
**Phenotype**	Pure	284	74	26.06	21.1–31.6
	Hybrid	62	27	43.55	31.0–56.7
**Hunting season**	2009	131	28	21.4	14.9–29.6
	2010	111	36	32.4	24.0–42.1
	2012	104	37	35.6	26.6–45.6
**Age**	Young	173	33	19.08	13.7–25.9
	Sub-adult	60	30	50.0	36.8–63.2
	Adult	112	37	33.04	24.6–42.6
**Domestic pigs**					
**Sex**	Male	91	78	85.71	76.8–92.2
	Female	117	105	89.74	82.4–94.4
**Breeding system**	Open	141	131	92.91	87.0–96.4
	Semi-open	35	33	94.29	80.8–99.3
	Closed	26	13	50.0	29.9–70.1
**Age**	Young	12	5	41.67	15.2–72.3
	Sub-adult	15	9	60.0	32.3–83.7
	Adult	128	120	93.75	87.7–97.1
	Old	51	48	94.12	83.8–98.8

**Table 2:** Sample size (*n*), seroprevalence (%) and 95% confidence intervals from phenotypically distinct wild boars (pure and hybrid) across the three hunting seasons (2009, 2010 and 2012). Differences between animals classified as pure and hybrids were significant (in grey) for the 2010 season (*p* = 0.01) and for all seasons (*p* = 0.006).

Hunting Season	2009 *n* = 131	2010 *n* = 109	2012 *n* = 104	Total Seasons *n* = 344
**Pure** *n* = 301	*n* = 25 20.6 (14–29)	*n* = 20 26 (17–37)	*n* = 29 34.5 (24.5–45.7)	*n* = 74 26.06 (21.05–31.57)
**Hybrid** *n* = 69	*n* = 3 30.0 (6.7–65)	*n* = 16 50 (32–68)	*n* = 8 40.0 (19.1–64)	*n* = 27 43.5 (30.9–56.7)
**Total Seropositives** *n* = 101	*n* = 28 21.37 (14.7–29.4)	*n* = 36 33 (24–42)	*n* = 37 35.6 (26.4–45.5)	*n* = 101 29.2 (24.5–34.3)
